# Effects of pain neuroscience education combined with blood flow restriction training on chronic postoperative pain following total knee arthroplasty: a randomized controlled trial protocol

**DOI:** 10.1186/s13018-026-07146-y

**Published:** 2026-08-20

**Authors:** Jipeng Xu, Peijie Sun, Jingfeng Yang, Yani Zhu, Yuanpeng Liao

**Affiliations:** 1 Department of Sports Medicine and Health, Chengdu Sport University, Chengdu, Sichuan China; 2 Institute of Sports Medicine and Health, Chengdu Sport University, Chengdu, Sichuan China; 3 Affiliated Sport Hospital of Chengdu Sport University, Chengdu Sport University, Chengdu, Sichuan China

**Keywords:** Chronic postsurgical pain, Total knee arthroplasty, Pain neuroscience education, Blood flow restriction training, Randomized controlled trial

## Abstract

**Introduction:**

Chronic postsurgical pain (CPSP) remains a major clinical challenge following total knee arthroplasty (TKA), with psychological maladaptation and persistent neuromuscular dysfunction considered key contributors to ongoing pain and disability. Pain neuroscience education (PNE) may improve maladaptive pain cognitions, whereas blood flow restriction training (BFRT) can enhance muscle function and induce exercise-related hypoalgesia. Although both interventions have shown potential benefits in chronic pain rehabilitation, no randomized controlled trial has investigated the combined efficacy of these two interventions in patients with CPSP after TKA. This study aims to determine whether combined PNE + BFRT produces greater improvements in pain and function in this population.

**Methods and analysis:**

This study is a single-blind, four-arm, parallel-group randomized controlled trial. A total of 148 participants will be randomly assigned (1:1:1:1) to the Control, PNE, BFRT, or combined PNE + BFRT group. PNE will be delivered in two structured sessions. BFRT involves a 12-week supervised low-load resistance training program. The primary outcome is the group-by-time interaction effect on KOOS₄ over time. Secondary outcomes include pain intensity (NRS), global perceived effect, pain distribution, psychological measures (HADS, PCS), physical performance (40-m fast-paced walk test, stair climb test, and 30-s chair stand test), and neuromuscular strength. Assessments will be conducted at baseline, post-intervention (12 weeks), and at 12-week, 24-week, and 36-week follow-ups. Data will be analyzed using linear mixed-effects models under the intention-to-treat principle.

**Ethics and dissemination:**

Ethics approval has been obtained from the Ethics Committee of the Chengdu Sport University, with permission number CDSUEC2026- 177. This study has been registered with the Chinese Clinical Trial Registry (ChiCTR2600125544). Written informed consent will be obtained from all participants prior to enrollment.

**Trial registration number:**

Chinese Clinical Trial Registry ChiCTR2600125544, registered on May 28, 2026.

**Supplementary Information:**

The online version contains supplementary material available at https://doi.org/10.1186/s13018-026-07146-y.

## Introduction

Knee Osteoarthritis (KOA) is a prevalent degenerative joint disease and a leading cause of disability worldwide [1, 2]. Total knee arthroplasty (TKA) is currently recognized as the preferred treatment for end-stage KOA [3]. Despite substantial improvements in pain and function following surgery, approximately 20% of patients continue to experience persistent moderate-to-severe pain lasting beyond three months postoperatively, commonly referred to as chronic postsurgical pain (CPSP) [4–6].

CPSP is not only associated with an increased risk of opioid dependence but also creates a substantial socioeconomic burden [7, 8]. Additionally, inadequately managed CPSP may precipitate comorbidities such as insomnia and hypertension [9], undermining surgical expectations and exacerbating doctor-patient conflicts [10]. Given the challenges and high cost of CPSP treatment, coupled with the poor efficacy of pharmacological prophylaxis [11–13], exploring efficient and safe treatment strategies is of significant clinical and societal urgency. The genesis and development of CPSP are multifactorial. Among these predictors, psychological factors are widely regarded as key predictors of CPSP [14–17]. Patients' emotional responses to pain profoundly influence their recovery trajectory, especially Pain Catastrophizing (PC), a maladaptive cognitive pattern, involving rumination, magnification, and helplessness, has been consistently confirmed as a potent, independent risk factor for CPSP [18–20].

Pain Neuroscience Education (PNE) has emerged as a prominent intervention for pain management. By emphasizing that pain is not strictly a direct signal of tissue damage, it effectively helps patients re-evaluate the nature of pain [21–25]. Clinically, PNE has proven effective in reducing pain intensity and significantly alleviating fear-avoidance beliefs, pain catastrophizing, and anxiety in chronic pain populations [22, 26, 27]. However, while PNE has beneficial effects on pain-related cognition and emotional factors, its effects on physical function and muscle performance remain relatively limited [28–30]. Therefore, PNE is optimally deployed as part of a multimodal treatment strategy, integrated with other interventions to elicit synergistic effects and deliver superior clinical outcomes.

Research indicates that therapeutic exercise can attenuate pain sensitivity in both motor and non-motor areas [31–33]. As exercise intensity and duration increase, the effect of inducing pain reduction becomes increasingly pronounced [34–36]. However, for patients with CPSP following TKA, engaging in prolonged high-intensity exercise may pose significant challenges. Blood Flow Restriction Training (BFRT) is a low-intensity training method that partially restricts arterial blood flow and blocks venous return by applying external pressure to the proximal end of the limb [37]. Emerging evidence suggests that BFRT can produce muscle hypertrophy, strength gains, and analgesic effects comparable to those achieved with high-intensity resistance training while imposing substantially lower mechanical stress on the joint [38–40]. Nevertheless, BFRT primarily targets physical and neuromuscular impairments and may not adequately address maladaptive pain-related cognition or fear-avoidant behaviors.

Accordingly, combining PNE with BFRT may offer a complementary rehabilitation strategy. PNE may enhance psychological readiness for rehabilitation by reducing pain-related fear, catastrophizing, and perceived threat associated with movement, whereas BFRT may promote functional recovery and pain relief. To date, no randomized controlled trial has investigated whether combining PNE and BFRT provides superior clinical benefits in patients with established CPSP following TKA. Therefore, this study is designed as a Randomized Controlled Trial to compare the effects of Control, PNE, BFRT, and combined PNE + BFRT on pain, psychological outcomes, physical performance, and knee-related function in patients with CPSP after TKA. We hypothesize that participants receiving the combined PNE + BFRT intervention will demonstrate greater improvements in knee-related function, pain intensity, psychological outcomes, and physical performance compared with those receiving either intervention alone or usual care.

## Method and analysis

### Study design

The study is designed as a single-center, four-arm, parallel-group, superiority randomized controlled trial. A total of 148 participants will be recruited from the Chengdu Sport University. All therapeutic interventions and data collection will be conducted at the School of Sports Medicine and Health, Chengdu Sport University.

Potential participants will be targeting individuals who underwent unilateral TKA at least one year prior. Eligible candidates will be contacted by telephone for preliminary screening. Those meeting eligibility criteria will be invited for an in-person baseline assessment and receive a comprehensive explanation of the study procedures. Written informed consent will be obtained before enrollment. A screening log will be maintained to document eligibility and reasons for exclusion. Participants will be consecutively recruited until the required sample size (n = 148) is achieved. Participants will be randomly assigned in a 1:1:1:1 ratio to one of four groups: the Control group, the PNE group, the BFRT group, or the PNE combined with BFRT group.

The study has received approval from the Ethics Committee of the Chengdu Sport University (Committee Reference Number: CDSUEC2026- 177) and is registered with the Chinese Clinical Trial Registry (Registration Number: ChiCTR2600125544). The trial will be conducted in accordance with the Declaration of Helsinki. Any protocol amendments will be promptly submitted to the Ethics Committee and updated in the trial registry. Given the low-risk nature of the interventions, an independent data monitoring committee will not be established. However, all adverse events will be recorded and monitored throughout the trial. Any serious adverse events (SAEs) will be immediately reported to the Ethics Committee.

**Fig. 1:**
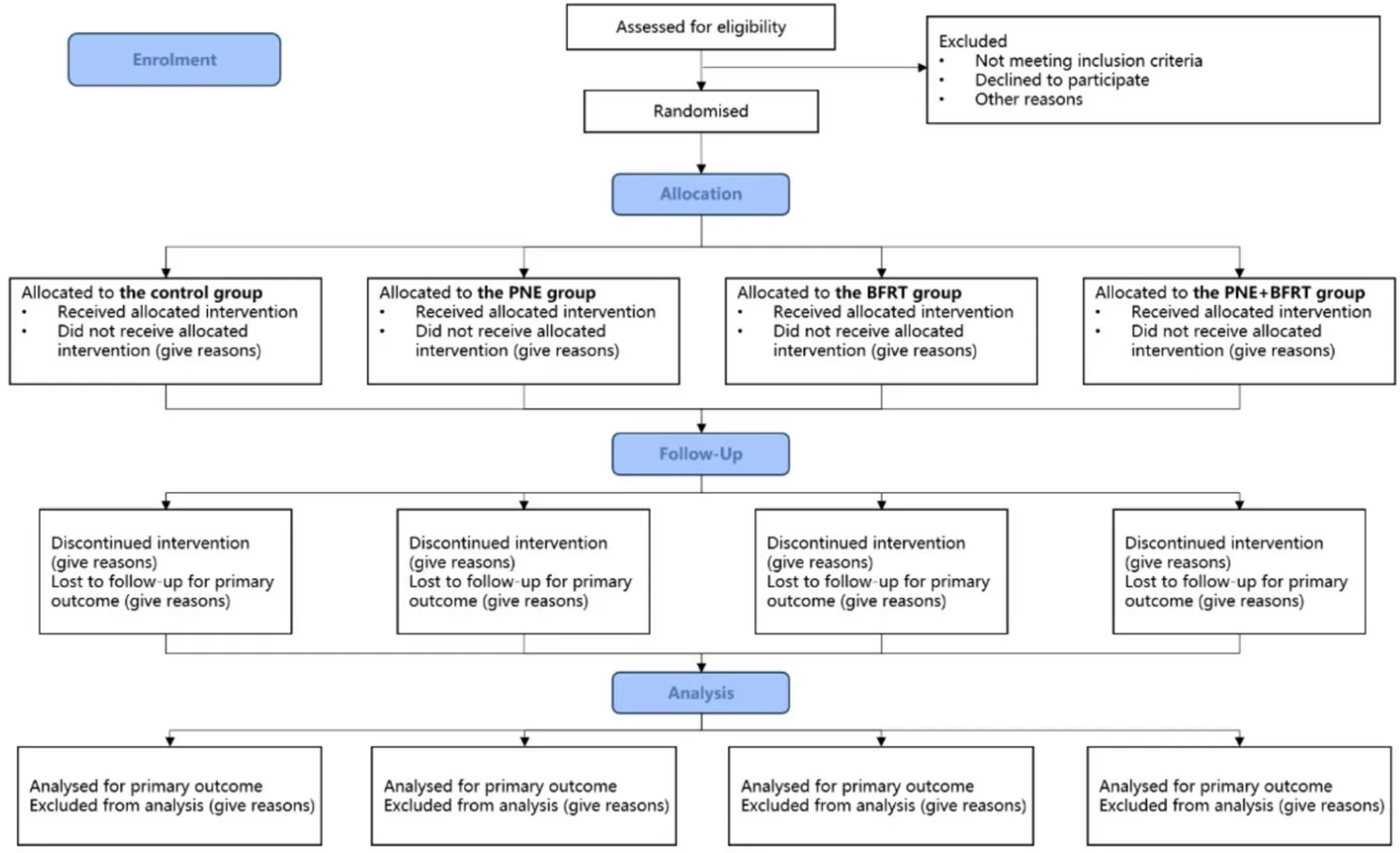
Study flow diagram

The study period will span from May 2026 to July 2029. Participant recruitment will occur between May 2026 and July 2027. Follow-up assessments will be completed by July 2029. The study protocol was developed in accordance with the SPIRIT 2025 Statement, as shown in Fig. 1. The timeline for participant enrollment, interventions, assessments, and follow-up visits is detailed in Table 1.

**Table 1:** Schedule of the process of trial

Period	Screening	Intervention			Follow-up		
Time point	Week 0	Week 0	Week 6	Week 12	Week 24	Week 36	Week 48
Eligibility	×						
Informed consent	×						
Allocation							
Control group		———————————————————————————————————————————————————					
PNE group		×	×				
BFRT group		———————————————————————————————————————————————————					
PNE + BFRT group		———————————————————————————————————————————————————					
KOOS₄		×		×	×	×	×
KOOS		×		×	×	×	×
NRS		×		×	×	×	×
GPE		×		×	×	×	×
Pain Extent and Distribution		×		×	×	×	×
HADS		×		×	×	×	×
PCS		×		×	×	×	×
40 m-FPWT		×		×	×	×	×
SCT		×		×	×	×	×
30CST		×		×	×	×	×
Muscle strength		×		×	×	×	×
Adverse events		————————————————————————————————————————————————————————————————————————————————————					
Dropout reasons		————————————————————————————————————————————————————————————————————————————————————					

PNE, Pain Neuroscience Education; BFRT, Blood Flow Restriction Training; KOOS, Knee Osteoarthritis Outcome Score; NRS, Numerical Rating Scale; GPE, Global Perceived Effect; HADS, Hospital Anxiety and Depression Scale; PCS, Pain Catastrophizing Scale; 40 m-FPWT, 40-m Fast-Paced Walk Test; SCT, Stair Climb Test; 30CST,30-s Chair-Stand Test.

### Inclusion criteria

Participants meeting all of the following inclusion criteria will be enrolled:

1. Adults who have undergone primary TKA for KOA with a postoperative period of ≥ 12 months.
2. Presence of persistent pain in the target knee for a duration of > 6 months.
3. Pain localized to the surgical site or its corresponding nerve distribution, with other potential causes excluded (e.g., prosthesis loosening, infection).
4. An average pain intensity score of ≥ 4 points on the Numerical Rating Scale (NRS) over the week preceding enrollment (0 indicates no pain, 10 indicates the most severe pain, and ≥ 4 indicates moderate to severe pain).
5. Stable vital signs, clear consciousness, and sufficient cognitive and linguistic ability to assess their own pain and cooperate in completing pain assessment scales.

### Exclusion criteria

Participants presenting with any of the following will be excluded:

1. Specific reasons for chronic pain, such as loosening and failure of the prosthesis, which requires revision surgery.
2. Secondary osteoarthritis associated with rheumatoid arthritis or sequelae of previous trauma.
3. Recent surgical history involving the index knee within 3 months or significant injury within the preceding 12 months.
4. Confounding pain patterns, defined as acute pain in areas other than the index knee during baseline testing.
5. Vascular contraindications, including a history of deep vein thrombosis, peripheral vascular disease, or active bleeding diathesis incompatible with compression training.
6. Concurrent medication or trial participation, specifically the use of opioid analgesics within 2 weeks or enrollment in other pain-related studies.
7. General ineligibility due to pregnancy, lactation, severe cognitive impairment, or inability to provide informed consent.

### Randomization and blinding

Participants will be randomly assigned in a 1:1:1:1 ratio to the Control, PNE, BFRT, or combined PNE + BFRT groups using a computer-generated randomization sequence created in SPSS 27.0 (IBM SPSS, Armonk, NY, USA) by an independent researcher not involved in recruitment, assessment, or intervention delivery. To ensure balance across groups while minimizing allocation predictability, permuted block randomization with randomly varying block sizes of 4 and 8 will be implemented. The allocation sequence will be concealed using a centralized, password-protected system accessible only to the trial coordinator, who will not be involved in participant recruitment, intervention delivery, outcome assessment, or data analysis.

Following completion of baseline assessment and confirmation of eligibility, the trial coordinator will assign participants to the corresponding intervention according to the pre-generated randomization sequence. Due to the nature of the interventions, blinding of participants and treating therapists is not feasible. Therefore, this trial will employ blinded outcome assessment and blinded statistical analysis. Outcome assessments will be conducted by trained assessors who are not involved in randomization or intervention delivery, and data analysts will remain blinded to group allocation throughout the study period. Participants will be instructed not to disclose their group allocation during assessment sessions to minimize the risk of unblinding. Statistical analyses will be performed using de-identified datasets with masked group codes to further reduce analytical bias.

### Sample size

The sample size was estimated using G*Power software (version 3.1.9.7, Franz Faul, Universität Kiel, Germany). The primary outcome is the KOOS₄ score (mean score of the Pain, Symptoms, Activities of Daily Living, and Knee-Related Quality of Life subscales) from the Knee Osteoarthritis Outcome Score (KOOS). Based on previous research, a 10-point improvement in KOOS₄ was considered the Minimum Clinically Important Difference (MCID), with an anticipated Standard Deviation (SD) of 15 points [41, 42]. The following parameters were utilized for the sample size calculation: Significance Level (α) = 0.05, Statistical Power (1-β) = 0.90, Effect Size (Cohen's f) = 0.34, and Number of Groups = 4. The calculations indicated that the minimum total sample size required to detect this effect was 128 cases. Considering an anticipated attrition rate of approximately 15% and to ensure equal and balanced allocation across four groups, the total sample size was increased to 148 participants, resulting in 37 participants per group. This approach enhances the stability and reliability of the study results, ensuring that the research objectives will be successfully met.

### Interventions

#### The control group

Participants allocated to the Control group will continue receiving their ongoing conservative management for persistent pain after TKA, including stable medication use, self-directed physical activity or exercise, and routine medical care when necessary. No structured PNE or supervised exercise intervention will be provided during the study period.

#### The PNE group

Participants allocated to the PNE group will additionally receive two one-hour PNE sessions delivered by a licensed physical therapist with formal training in pain neuroscience education [28]. The primary goal of PNE is to alter maladaptive pain cognitions, helping participants to reconceptualize their pain and enabling them to better self-manage their symptoms [43]. The first session (Week 0) will introduce core concepts of pain neuroscience, including peripheral and central sensitization, hyperalgesia, and the distinction between tissue damage and pain perception. Mechanisms underlying CPSP following TKA will be explained using patient-centered language and visual aids. The second session (Week 6) will reinforce previously introduced concepts, address participants’ pain-related questions, facilitate discussion of pain management experiences, and further strengthen participants' cognitive understanding and coping abilities regarding their own pain. Participants will receive a concise information booklet summarizing the PNE topics. The detailed curriculum and learning objectives for each session are outlined in Supplementary Appendix 1. Attendance at both PNE sessions is considered complete adherence to the core intervention, whereas attendance at only one session is considered partial adherence and failure to attend either session is considered non-adherence.

#### The BFRT group

Participants in the BFRT group will additionally undergo a 12-week supervised BFRT program conducted twice weekly for a total of 24 sessions [28, 44]. Before the intervention, participants will complete three familiarization sessions separated by at least 48 h to standardize exercise technique and minimize learning effects [45]. The individualized Arterial Occlusion Pressure (AOP) will be determined using a Doppler ultrasound probe (DV-600; Marted, Brazil) in the supine position [44]. Detailed procedures for AOP measurement are provided in Supplementary Appendix 2. The training intensity will be anchored to the participant's 1-repetition maximum (1-RM) for leg press and knee extension exercises assessed using standard resistance equipment (Nakagym, São Paulo, Brazil). The 1-RM assessment protocol is detailed in Supplementary Appendix 3 [44, 46]. The intervention will follow a progressive overload model. The first week served as an adaptation phase, employing a training intensity of 20% 1-RM, completed in 4 sets of 15 repetitions each. Starting from Week 2, the training intensity was increased to 30% 1-RM, and from Week 5, the number of sets per training session was increased to 5. Inter-set rest intervals will be standardized at 1 min. During training, cuff pressure will be set at 70% of the individualized AOP, and the cuff will be inflated immediately before the first set and deflated after completion of the final set [44].

To maintain training load stability and ensure BFRT efficacy, both the 1-RM and AOP will be reassessed every 4 weeks to adjust training loads and cuff pressure based on updated values. Throughout the intervention, adverse events and participant discomfort will be continuously monitored.

#### The combined intervention group (PNE + BFRT)

In addition, participants allocated to the combined intervention group will receive both PNE and BFRT according to the same protocols described above. PNE sessions will be delivered at Weeks 0 and 6, while BFRT will be conducted twice weekly throughout the 12 weeks. On weeks when both interventions occur, PNE sessions will precede exercise training to avoid fatigue-related interference.

Participants in all groups will be instructed to maintain their existing medications and conservative management strategies throughout the study period whenever possible.

Any concomitant treatments, including analgesic use, physiotherapy, injections, or additional rehabilitation interventions, will be recorded during follow-up.

### Outcomes

Patients with CPSP following TKA often experience not only pain but also difficulties with activities of daily living (ADL). Since our goal is to both alleviate pain and improve physical function, we have selected the following measures as outcome indicators. Outcome assessments will be conducted at baseline, post-intervention (12 weeks), and follow-up at 24, 36, and 48 weeks [28].

### Primary outcome

#### KOOS_4_

The primary outcome is the between-group difference in longitudinal change in KOOS₄ across all assessment time points. KOOS_4_ was selected as the primary outcome measure because persistent CPSP after TKA affects not only pain intensity but also broader dimensions of knee-related symptoms, activities of daily living, and quality of life, making patient-centered assessment essential when evaluating treatment outcomes. It is one of the most widely used and validated assessment tools in patients undergoing TKA [28].

The KOOS₄ is calculated as the mean of four KOOS subscales (Pain, Symptoms, Activities of Daily Living, and Knee-Related Quality of Life), excluding the Sport/Recreation subscale. Scores range from 0 to 100, with higher scores indicating better knee-related function [47, 48]. Based on prior literature, A between-group difference of 10 points is considered the MCID and will be used to interpret the clinical relevance of observed effects [42].

#### Secondary outcomes

Secondary outcomes will include patient-reported, psychological, physical performance, and neuromuscular measures.

### Patient-reported outcome measures (PROMs)

#### KOOS

To assist the clinical interpretation of the primary outcome, we will further conduct separate analyses on all five subscales of the KOOS (Pain, Symptoms, Activities of Daily Living, Sports and Recreation, and Knee-Related Quality of Life) [49]. This granular analysis provides a comprehensive clinical basis for interpreting functional recovery across specific domains.

#### Numerical rating scale (NRS)

Pain intensity will be quantified using the NRS, ranging from 0 (no pain) to 10 (worst possible pain). We will assess the average daily pain intensity in the target knee over the preceding week, as well as the maximum intensity for resting pain (daytime and nighttime) and activity-evoked pain (during walking and stair climbing). A change of ≥ 2 points is considered clinically relevant [28].

#### Global perceived effect (GPE) scale

The GPE Scale is a simple and commonly used tool for assessing patients' subjective therapeutic efficacy, with excellent reliability [50]. Participants will rate their overall improvement by answering the prompt: 'How do you feel about your symptoms and overall condition now compared to before treatment?" [28]. Scores are based on a 7-point Likert scale, ranging from 1 (significant improvement) to 7 (significantly worse), with higher scores indicating more severe symptoms.

#### Pain extent and distribution

Participants will mark their habitual pain areas on a standardized anatomical body chart, and the pain pattern is classified using a knee pain map to visually assess changes in pain distribution, providing "spatial dimension" information beyond pain intensity and offering a more comprehensive reflection of pain improvement.

### Psychological outcomes

#### Anxiety and depression

The Hospital Anxiety and Depression Scale (HADS) will be employed to assess emotional status. It comprises two subscales: Anxiety (HADS-A) and Depression (HADS-D), each ranging from 0 to 21 points. Scores ≤ 7 indicate normal status, 8–10 represent borderline abnormal status, and scores ≥ 11 denote abnormal levels [51].

#### Pain catastrophizing

The Pain Catastrophizing Scale (PCS) will serve to measure maladaptive thinking patterns related to pain, and has demonstrated good reliability and validity across diverse cultural and clinical populations [52]. It includes 13 items across three dimensions: rumination, magnification, and helplessness. Each item is rated from 0 (never) to 4 (always), yielding a total score range of 0 to 52. A total score ≥ 30 indicates clinically significant pain catastrophizing tendencies.

### Physical performance

Objective physical function will be assessed using a battery of performance-based tests: the 40-m fast-paced walking test, stair climbing test, and 30-s chair-stand test [53].

#### 40-m fast-paced walk test (40 m-FPWT)

Participants walk 40 m along a flat, unobstructed straight path at their maximum safe speed. The total time is recorded to calculate walking speed (m/s).

#### Stair climb test (SCT)

The time required to safely ascend and descend a flight of nine steps is recorded. Use of handrails or assistive devices is permitted if necessary.

#### 30-s chair-stand test (30CST)

Participants are instructed to perform as many full sit-to-stand cycles as possible within 30 s. Full hip and knee extension is required for each repetition. Assistive devices are permitted if the participant is unable to complete the task independently [54, 55].

### Neuromuscular assessment

#### Muscle strength

As described in the intervention protocol, the 1-RM for leg press and knee extension will be assessed using standard resistance equipment (Nakagym, São Paulo, Brazil). Additionally, the maximum isometric contraction strength of the quadriceps, hamstrings, and gluteus medius will be measured using a handheld dynamometer (Lafayette Manual Muscle Tester, USA). The hamstring-to-quadriceps (H: Q) ratio will be calculated to evaluate muscle strength balance [28].

### Statistical analysis

All statistical analyses will be conducted using SPSS version 27.0 (IBM Corp., Armonk, NY, USA) by statisticians blinded to group allocation. All tests will be two-tailed with a significance level set at *P* < 0.05. Baseline characteristics will be summarized using descriptive statistics. Continuous variables will be presented as mean ± standard deviation (SD) or median (interquartile range), depending on data distribution. Categorical variables will be expressed as frequencies and percentages. All efficacy analyses will follow the intention-to-treat (ITT) principle. The primary analysis will focus on evaluating whether the longitudinal response trajectory of the combined PNE + BFRT group differs from those of the comparison groups over time. To address this objective, linear mixed-effects models (LMMs) will be used for repeated-measures analyses of the primary and secondary outcomes. The primary inference of interest will be based on the group-by-time interaction effect, which reflects differential changes in outcome trajectories between intervention groups throughout the study period. To reduce the risk of type I error inflation, KOOS₄ will be considered the sole confirmatory primary outcome, while all other outcomes will be treated as secondary or exploratory. If a significant group-by-time interaction is identified for KOOS₄, post-hoc pairwise comparisons with Bonferroni correction will be performed. Secondary outcomes will be analyzed using similar mixed-effects modeling approaches. Estimated marginal mean differences with 95% confidence intervals will be reported where appropriate. Missing data will be assumed to be missing at random and handled using maximum likelihood estimation within the mixed-effects modeling framework. A supplementary per-protocol analysis, including participants with at least 80% adherence to the intervention, may also be conducted.

### Data management

To ensure the standardization and security of research data, all study data will be entered into a secure, password-protected electronic database accessible only to authorized research personnel. Double data entry and periodic data verification will be conducted to ensure accuracy. Each participant will be assigned a unique identification code to maintain the confidentiality of personal information. In accordance with institutional data retention policies, all physical and electronic records will be archived for five years following the publication of results, after which they will be securely destroyed.

### Adverse events

Safety monitoring will be conducted continuously. Any adverse events reported by participants or observed by staff will be recorded, detailing their severity and potential relationship to the intervention. All adverse events and reasons for withdrawal will be documented in detail and reported to the Ethics Committee and the clinical trial registry, and will be analyzed in future studies.

### Patient and public involvement

While participants will not be directly engaged in the study design, their preferences regarding treatment burden and pain management will be considered throughout all stages of the research. Study findings will be shared with participants upon conclusion.

## Discussion

KOA is the most common form of OA, accounting for approximately 85% of the total disease burden. Its typical clinical manifestations include joint pain, stiffness, and functional impairment, which compromise joint stability and, in severe cases, may lead to loss of mobility, posing a major public health challenge [1, 2, 56]. TKA is an effective treatment option for patients with moderate-to-severe KOA. However, 10–34% of patients report CPSP after primary TKA [57]. CPSP not only hinders postoperative recovery but may also lead to disability, diminish quality of life, and place a substantial burden on patients, families, and the healthcare system. Therefore, identifying effective interventions to alleviate pain and improve functional outcomes in patients with CPSP following TKA is of great clinical significance.

The development and progression of CPSP are influenced by multiple factors, among which psychological factors and chronic inflammatory responses are considered major contributors [58–62]. To avoid anticipated pain, patients often limit or avoid physical activity, a behavior that may ultimately lead to functional decline, muscle atrophy, and heightened pain sensitivity. Decreased muscle strength and the persistent accumulation of abnormal or elevated biomechanical loads may cause joint or tissue damage, further exacerbating pain and ultimately forming a vicious cycle of pain, fear, avoidance, functional loss, and renewed pain [63–66]. PNE, as a psychological intervention designed to reshape patients' perceptions of pain and coping strategies, positively influences pain experiences by explaining the biological and physiological mechanisms involved in pain. It has demonstrated small to moderate efficacy in chronic musculoskeletal pain [23, 67–69]. Among patients with persistent chronic pain one year after TKA, PNE has been shown to have clinical significance for pain relief, but its role in improving function remains relatively limited [28]. BFRT, a low-intensity exercise modality that applies external pressure to the proximal limb to partially restrict arterial blood flow and occlude venous return, has attracted significant interest for its exceptional efficacy in inducing muscle adaptation and analgesia. The pain-relieving mechanism is believed to be associated with metabolic stress–induced endogenous opioid release and inflammatory modulation [38, 70–72]. Although BFRT demonstrates promising clinical prospects in various chronic pain and postoperative rehabilitation, no study has yet systematically evaluated its efficacy in patients with CPSP following TKA. Given the potential complementary mechanisms between PNE and BFRT, it is reasonable to hypothesize that combining PNE with BFRT may produce synergistic effects in pain reduction and functional improvement, potentially offering superior clinical benefits for patients with CPSP following TKA.

To the best of our knowledge, this is the first study to compare the effects of PNE and BFRT in patients with persistent chronic pain one year after TKA and to assess whether combining both approaches yields superior clinical outcomes. Should the final results demonstrate that the combined group significantly outperforms either single intervention group, it would strongly support the clinical application of this integrated rehabilitation strategy. If effective, the combined intervention may provide an integrated non-pharmacological rehabilitation strategy targeting both psychological and physical contributors to CPSP following TKA. It is noteworthy that patients undergoing TKA typically experience a natural trend toward pain resolution within three months to one year post-surgery [73], which may interfere with the interpretation of short-term intervention efficacy. Therefore, this study specifically recruits individuals who continue to experience CPSP one year postoperatively, aiming to minimize the influence of natural recovery and improve the accuracy of treatment-effect evaluation.

However, this study still has several limitations. Firstly, the relatively long follow-up period may increase the risk of participant dropout, potentially affecting the representativeness and the statistical power of the final sample. To mitigate this issue, strategies such as regular communication, progress feedback, and incentive-based engagement will be implemented during the study to enhance participant compliance and ensure data completeness. Secondly, this study primarily focuses on validating clinical efficacy and does not delve into the potential molecular mechanisms underlying the synergistic effects of PNE and BFRT. This direction warrants further exploration in future research. Finally, the four groups differ in therapist contact and supervision, and no attention-matched comparator is included. Consequently, non-specific effects related to therapist attention, supervision, treatment expectations, and therapeutic interaction cannot be completely separated from the specific effects of the interventions. Although intervention exposure and protocol deviations will be documented, the findings should be interpreted as the effects of the respective intervention strategies as delivered. Future studies incorporating attention-matched comparators are warranted to better isolate specific intervention effects.

## Supplementary Information

Below is the link to the electronic supplementary material.

Supplementary Material 1

Supplementary Material 2

## Data Availability

Data are available in a public, open access repository. Data are available on reasonable request.
