# Supplementary material for: Effects of pain neuroscience education combined with blood flow restriction training on chronic postoperative pain following total knee arthroplasty: a randomized controlled trial protocol
Source: J Orthop Surg Res. 2026 Aug 20;21:555. doi: 10.1186/s13018-026-07146-y (PMC13599210; doi:10.1186/s13018-026-07146-y)
Supplement: Supplementary file 2 [file 13018_2026_7146_MOESM2_ESM.docx]

**Supplementary Materials:**

**Appendix 1: PNE Curriculum Content**

The First: Reconceptualizing Pain (Baseline)

1. Basic Pain Understanding: Clearly distinguish between acute and chronic pain concepts. Emphasize that chronic pain is not a direct reflection of tissue damage but rather a product of the nervous system's "hyperprotection," not necessarily linked to the extent of tissue injury.

2. Chronic Pain Triggers: Systematically analyze the mechanisms of chronic pain development across multiple dimensions, including individual factors (e.g., gender, age), psychological factors (e.g., catastrophizing thoughts about pain, anxiety, depression), and physiological factors (e.g., central and peripheral sensitization, inflammatory responses).

3. Neural Sensitization Analysis: Explains that neural sensitization refers to an abnormally heightened responsiveness of the central or peripheral nervous system to stimuli. This can result in significant pain triggered by minor stimuli, or even harmless stimuli, and may be accompanied by pain spread and increased intensity.

4. Coping Strategies: Participants will be encouraged to adopt proactive coping strategies based on an improved understanding of pain mechanisms and to reduce unnecessary avoidance behaviours related to pain. The therapist will discuss how excessive fear of movement and avoidance of meaningful activities may contribute to ongoing disability and reduced confidence. Participants will be encouraged to gradually resume meaningful daily activities according to their individual abilities and circumstances, while recognizing that gradual engagement in activity does not necessarily indicate tissue damage or worsening of the underlying condition.

The Second Session: Consolidation and Application (Week 6)

1. Core Review from Session 1: Reaffirmed pain's essence as a "protective warning signal," reiterated that chronic pain is not directly tied to the extent of tissue damage. It also highlighted the central role of neural sensitization in maintaining chronic pain.

2. Identifying " Distinguishing 'Danger Signals'" from "Safety Signals": Clearly differentiate "danger signals" that perpetuate pain (e.g., exercise fear, negative cognition, emotional suppression) from "safety signals" that facilitate pain relief (e.g., good physical function, positive social support, regular physical activity).

3. Application of Brain Plasticity: Explains the nervous system's plasticity, demonstrating that through cognitive restructuring and progressive exercise interventions, normal protective neural functions can be gradually restored. This reduces unnecessary pain perception, ultimately achieving pain relief and functional improvement.

4. Practice and Feedback: The therapist will review participants’ experiences since Session 1, address pain-related questions, and provide feedback regarding the application of the pain neuroscience concepts discussed during the intervention. Discussion will focus on how participants understand and respond to their pain in daily life, including their pain-related beliefs, perceived threat, emotional responses, activity avoidance, and functional goals. Participants will be encouraged to apply the principles of pain reconceptualization and proactive pain self-management to their daily lives. Individualized discussion will be used to help participants identify practical ways to gradually resume meaningful activities and reduce unnecessary avoidance according to their own abilities and circumstances. The therapist will provide feedback and clarification based on participants’ experiences, without providing a separate structured exercise program.

**Appendix 2: AOP Assessment Protocol**

To ensure standardized occlusion, resting AOP is measured as follows:

1. Participants lie in a supine position in a quiet, temperature-controlled room.

2. A vascular Doppler probe (DV-600; Marted, São Paulo, Brazil) is placed over the posterior tibial artery to identify the auscultatory pulse.

3. The pneumatic cuff (175 mm*920 mm) is positioned at the most proximal portion of the thigh (inguinal fold).

4. The cuff is gradually inflated until the Doppler pulse signal is completely interrupted. This value is recorded as the AOP (100% occlusion).

**Appendix 3: 1-RM Assessment Protocol**

1. Warm-up: Participants perform two sets of low-intensity repetitions with a 2-minute rest interval.

2. Testing: The load is gradually increased to identify the maximum weight the participant can lift for a single repetition with proper form.

3. Constraints: A maximum of five attempts is permitted per session to avoid fatigue, with a 3-minute rest interval between attempts.
